# Justifiableness of Exploratory Operations1Read before the Warren County (Pa.) Medical Society, December, 1903.

**Published:** 1904-04

**Authors:** Eugene A. Smith

**Affiliations:** Buffalo, N. Y.


### Justifiableness of Exploratory Operations.[1]

By EUGENE A. SMITH, M. D., Buffalo, N. Y.

AT THE bedside of the patient the physician becomes the exponent of the art rather than the science of medicine. In the effort to diagnosticate the disease in a given case he may apply scientific methods to the best advantage and in the most approved way; yet time, an operation, or a post-mortem will show more or less than he diagnosticated, or, perhaps, that he was quite at fault in regard to some complication or unusual condition not at all expected. The mathematician solves his problem with certain given data, and thereafter he can name his formulæ to solve all problems in which such data are the factors. The physician cannot do this; he must always deal with an element of probability or uncertainty, arising from the many influences modifying the etiological factors of disease and the varying factors of susceptibility and resistance in the patient. Under seemingly quite similar conditions in given patients he cannot be absolutely certain of like diagnoses.

In the practice of medicine, men who deal most successfully with this element of probability or uncertainty are said by those of us who admire their ability, to be possessed of the so-called clinical sense. They are men who fail less often than the rest of us. They succeed more often by reason of acute observing power, experience and a tactful judgment, rather than from scientific knowledge. They may be greater scientists; they are always greater artists. As optimists we may declare that the great advances made in recent years in the science of medicine have made the art of medicine less uncertain, but we must admit that the art of medicine still halts, stumbles and limps in the footsteps of scientific medicine, and will do so till the end of time, no matter what advances the future may have in store.

It is my purpose to talk tonight of one of the tendencies creeping into the practice of medicine, going with and due to the

1. Read before the Warren County (Pa.) Medical Society, December, 1903.

advances of medicine, or to speak more specifically, surgery. This tendency is fostered by the element of uncertainty in the practice of medicine already mentioned, and the comparative safety of surgical work due to modern asepsis and antisepsis. I speak of exploratory operations which have become a reproach to surgery in so far as they make us less careful in diagnosis, and more dependent upon the knife to determine pathological conditions. If this evil grows, it may some day be said that the forefathers in medicine, with fewer scientific means of clinical study, diagnosticated more correctly at the bedside than we who have modern pathology and modern laboratory methods to aid us in interpreting clinical facts.

To all of us it should be a gratification and a pride to make correct diagnoses. Outside and above the considerations of a selfish or pecuniary nature which reward the physician for correct diagnostic work, medicine in a broad sense is elevated, there is lessened opportunity for criticism of the profession, and quackery and ignorance are confounded. The more exact the diagnosis the more the sufferer benefits, and in assuming the responsibility of his case we are in duty bound to give him our best judgment after close study—first, to determine his illness, and, second, to treat it. To satisfy our own consciences, therefore, we must make our patients the unconscious critics and censors of our work in the light of our results.

The conscientious diagnostician realises that his error is dangerous to his patient. The erring diagnostician becomes a blundering clinician and is dangerous to the community when he is too obtuse to profit by his errors, and has, withal, the courage, or, in this sense we may better say, the hardihood to stand upon his convictions. Such diagnosticians do not take their gross errors with sufficient humiliation and self-reproach. They ignore the minor, less disastrous ones. They consider the patient and his friends unreasonable because a professional mistake, perhaps seriously affecting the patient, is discussed by the sufferer and his friends without equanimity and with criticism. Such medical men are prone to recommend or make use of the exploratory operation, and they advise operation in the expectation of finding something wrong which they are hopeful surgery can correct. Built on such a basis, their views of operative work are justly regarded with suspicion by the profession, although they are plausibly put to use in duping the laity. We may drop the discussion of this class of men with the observation that they are certain to retrograde unless, happily, they see the error of their ways and develop a conscience and sense of responsibility.

The art of medicine progresses with the science of medicine. Let us welcome every advance which makes diagnosis more accurate, treatment more beneficial, and prognosis more exact. Following Lister, the pioneers in antisepsis and asepsis have pointed the way to modern operative work with its comparative freedom from the old time danger of sepsis. Laboratory and clinical methods have also developed until diagnosis is far more certain in the practice of the willing worker, anxious to make use of the fullest opportunities to interpret the signs and symptoms of disease. When diagnostician and operator working, alone or together, strive to make the knife the means of curing disease and not a means for diagnosticating it, exploratory operations of objectionable type will seldom occur.

Before entering upon a closer discussion of exploratory operations, the investigating feature of all operations should be discussed. All operations are more or less exploratory, dependent upon the element of probability or uncertainty already discussed. A simple operative indication may lead the surgeon into operative problems far more extensive and serious than were expected. All surgeons usually lay out instruments and consider procedures which may be necessary for probable complications in a case about to be operated upon, although these complications are not diagnosticated nor expected. The surgeon who best plans his work to meet all contingencies before operating is the best surgical strategist. The pair of scissors, the hemostat, the needle and catgut which Robert Morris affects, were sufficient on one occasion when I saw him do a nephropexy, but he had to pause in operating, awaiting the sterilisation of a pair of bone forceps to remove the coccyx for coccygodynia in the same patient. The exploratory element in operations is typically seen in many cases of obstruction of the bowel in which the cause of the obstruction is unknown. Here abdominal section is undertaken to relieve the obstruction. The operation made necessary by the cause of the obstruction follows the exploratory opening of the abdomen, and may be any one of several standard operations depending upon whether the cause is within the bowel, without the bowel, or in the bowel tissue itself.

No one questions the advisability, the justifiableness, or the benefit of operations distinctly indicated for the relief of conditions only to be treated surgically, when the exploratory element is of minor importance in the diagnosis. When, however, the exploratory element in an operation becomes more and more pronounced, and the question of benefit to be derived from the operation becomes more and more problematical, the decision as to the advisability and justifiableness becomes more and more difficult.

From the ethical point of view we may divide exploratory operations into two classes, the first of which we may name justifiable exploratory operations, and the second unjustifiable exploratory operations. The justifiable exploratory operation is done on a case in which diagnosis or surgical indication is obscure, or in which both are obscure, but which promises possibility of benefit to the patient, and is indicated to relieve suffering or to avert impending danger. Obscure traumatisms, deeply placed tumors, septic conditions, and chronic conditions resulting from these causes, lead us often to justifiable exploratory work. In a general way we may instance cerebral or abdominal visceral injuries, septic peritonitis, either local or general, or tumors in the cavities of the body, as conditions which we often vaguely diagnosticate as to pathological cause, correctly diagnosticate as to operative need, and successfully attack when exploratory incision reveals the exact diagnosis.

When in these conditions a cause of dangerous disease is found which can be remedied or removed, success caps the popular and professional verdict of approval of the exploration, and the physician's judgment is commended. It does not follow that the physician is to be condemned for having done an unjustifiable exploratory operation, because at times hopeless conditions are found upon exploration, such as inoperable tumors. Such operations border on the unjustifiable, and are at best useless, or unwise. They are unjustifiable if careless diagnostic work was lacking before resort was made to the knife; in other words, the knife was unjustifiably used if diagnosis could have been made by careful clinical and laboratory methods. An error in judgment may lead to an unwise justifiable exploratory operation. For example, we may open the abdomen to find the cause of acute general peritonitis, the condition of the patient being such that his ability to survive operation is in question. Recently I lost a patient just after operation who died from acute general peritonitis, which I found was due to a hopeless condition of suppurative pancreatitis. In such a case a post-mortem to find the cause of disease would have been wiser than my antemortem operation. Death on the table or shortly after operation is a reproach to surgery and the surgeon's judgment. The man who declines to operate in dubious cases has fewer failures to his discredit, but, in my opinion, it is better to err in the patient's behalf in many cases in which it is difficult to decide whether operation is justifiable or not. Occasionally, seemingly moribund patients will rally under stimulating treatment after operation to relieve a condition which was necessarily fatal without operation.

At times we lean too far away from exploratory work in our attitude of disapproval of exploratory operations. Recently I operated, on a Friday evening, for acute general peritonitis, the history of the patient's illness dating back to the previous Tuesday morning when he was kicked by a horse in the abdomen. During Tuesday, Wednesday and Thursday he had abdominal pain without fever or rise of pulse. Alarming illness began Friday morning after cathartics were given. I found that bowel perforation due to sloughing of a portion of the wall of the ileum, no larger than a five-cent piece, was the cause of peritonitis. He died. Earlier exploratory operation might have saved his life. Circumstances of time, place and conditions, a good or a bad history in a case, and other factors may determine a justifiable exploratory operation, when under different circumstances the same case operated upon would come under the head of unjustifiable exploratory operations.

Battle field surgery deals with exploratory operations in the cavities of the body, mainly because of hemorrhage. When necessity does not compel, exploratory surgery is referred to base hospitals for better facilities and greater safety. Advanced age and infancy are deterring factors in exploratory work, yet an infant may be saved by abdominal section for bowel obstruction if the cause be remediable as, for example, intussusception. In the unconscious patient with vague history of skull injury, localising signs pointing to cerebral compression call for exploratory trephining. This does not hold true in the unconscious patient with arteriosclerosis and evidences of tertiary syphilis. The pathological condition in appendicitis is generally not to be diagnosticated by the clinical signs and operation, in this disease, has always an exploratory feature in acute cases.

A patient seen at midnight in the country suffering from appendicitis even though it seems to be subsiding is safer after immediate appendectomy than under conservative treatment. The same patient in a hospital ward might be treated conservatively twelve or twenty-four hours, or longer, or, perhaps, until the attack subsided, awaiting the quiescent period for operation. Exploratory needle puncture of the thorax is a procedure scarcely to be dignified by the name of an operation. But when such puncture shows the presence of fluid, the exploring needle decides the operation, either aspiration for hydrothorax or thoracotomy for pyothorax. Exploratory puncture is entirely justifiable and should be urged in the dubious cases in which differential diagnosis between pulmonary consolidation and pleural effusion is obscure. In my experience in consultation work, it is a diagnostic procedure too often neglected or postponed, with serious disaster resulting to the patient, especially in cases of empyema diagnosticated to be slowly resolving pneumonia.

Justifiable exploratory operations upon the contents of the skull are undertaken more and more frequently as shown by the reports in medical journals. Careful diagnostic work to determine the cause and location of compression is rewarded by successful trephining to relieve clot due to extradural and subdural hemorrhage, depressed bone abscess or tumor. Properly selected cases of epilepsy are benefited or cured by such operation. Differential diagnosis between cortical and subcortical or basilar lesions producing intracranial pressure is often extremely difficult, and with syphilis and traumatism excluded the differential diagnosis of cause may be also a problem. In the light furnished by the history and careful diagnostic work looking to cerebral localisation of the disease, exploratory trephining is justifiable and may be life-saving. In contradistinction to justifiable exploratory operations, unjustifiable explorations are done on cases in which diagnosis is obscure or the surgical indication is weak, and which give small promise of benefit to the patient by averting danger or lessening suffering. From the ethical point of view the element of hope in the exploratory work is the redeeming feature, which places this class of operations on a plane a trifle above operations which are altogether unjustifiable. In the hands of the careless diagnostician unjustifiable exploratory operations are unnecessary or useless, or dangerous; as, for example, exploratory incision of an aneurism. In the hands of the over-bold operator they are experimental or rash. In the hands of the unscrupulous operator they are reckless, and under stress of pecuniary temptation they are altogether unjustifiable and they may be criminal. If we may so speak their highest recommendation is the euthanasia they too often give the victims.

Speaking of diagnosis of disease within the abdominal cavity, some men speak of the abdomen as a "grab-bag." The man who regards the abdomen as a cavity to open and explore upon any and every opportunity, differs essentially from the man who regards the abdomen as a cavity to percuss and palpate after clinical study of the patient, with a view to decide what definite diseased condition needs medical treatment or surgical operation. The first man does unjustifiable exploratory operations. The second, as a rule, is justified when he does an exploratory operation. Asepsis lessens danger in surgical work. We must not let asepsis weaken surgical conscience and surgical judgment. The surgeon is not complimented who is called dextrous, whose operative technic is said to be perfect, but whose judgment is called poor. We might say of him that a good carpenter was spoiled to make a poor surgeon.

In conclusion, we may sum up much that is discursive in a paper of this kind by stating that exploratory operations are unjustifiable and are not to be recommended in so far as they are undertaken without careful clinical study of the patient and without painstaking use of the laboratory aids within our reach, and when the surgical indication is not dependent on trouble in the region to be attacked. An error in judgment may lead a conscientious surgeon to make an occasional operation properly classed with unjustifiable exploratory operations. Habitual recourse to exploratory operations by a surgeon must result in many unjustifiable operations, and becomes an error in ethics.

1018 Main Street.

